# Reliable management of post-esophagectomy anastomotic fistula with endoscopic trans-fistula negative pressure drainage

**DOI:** 10.1186/1477-7819-12-240

**Published:** 2014-07-30

**Authors:** Yi-Nan Liu, Yan Yan, Shi-Jie Li, Hui Liu, Qi Wu, Li-Jian Zhang, Yue Yang, Jin-Feng Chen

**Affiliations:** 1 Key Laboratory of Carcinogenesis and Translational Research (Ministry of Education), Department of Thoracic Surgery II, Peking University Cancer Hospital & Institute, 100142 Beijing, People’s Republic of China; 2 Key Laboratory of Carcinogenesis and Translational Research (Ministry of Education), Endoscopy Center, Peking University Cancer Hospital & Institute, 100142 Beijing, People’s Republic of China; 3 Beijing Pharma and Biotech Center, 100193 Beijing, People’s Republic of China

**Keywords:** anastomotic fistula, esophagectomy, endoscopic management

## Abstract

**Background:**

A gastroesophageal anastomotic fistula remains a potentially life-threatening post-esophagectomy complication. To promote fistula closure, we developed a modified endoscopic method of trans-fistula drainage with persistent negative pressure. In this study, we aimed to evaluate the efficacy of this endoscopic therapy.

**Methods:**

Between June and November 2013, five male patients with post-surgical esophageal leakages who had undergone trans-fistula drainage therapy were treated with the modified endoscopic trans-fistula negative pressure drainage (E-TNPD) method. We placed a nasogastric silicone tube into the paraesophageal cavity through the fistula and accomplished drainage of the infected effusion with continuous negative pressure, resulting in shrinkage of the para-anastomotic cavity and eventual fistula closure. We withdrew the trans-fistula drainage when there were no signs of leakage, as confirmed by esophagography. Final closure was confirmed by esophagography before the patient was allowed to begin oral intake.

**Results:**

E-TNPD was successful in all five patients. The median duration of drainage until tube removal was 34 days (range: 18 to 81 days). The duration for Cases 1 to 4 was 18 to 28 days. Case 5 suffered from multiple separate leaks at the anastomotic site and the gastric conduit. Complete restoration was achieved in 81 days for this patient. We found that in general, the earlier that trans-fistula drainage was established, the shorter the duration of hospitalization until complete defect closure.

**Conclusions:**

E-TNPD provided reliable and convenient management of post-surgical gastroesophageal anastomotic fistula and esophageal perforation. This method promoted fistula closure and prevented unnecessary repeated endoscopic examinations, extra equipment and expense.

## Background

Esophageal cancer is the eighth most common cancer and the sixth leading cause of death from cancer worldwide [1]. Gastroesophageal resection combined with multimodal therapy regimens is the standard treatment for resectable esophageal cancer. However, an anastomotic fistula remains a potentially life-threatening complication post-esophagectomy, with an incidence ranging from 3% to 25% [2-5]. Esophageal leakage can result in mediastinitis and subsequent sepsis and can lead to prolonged hospitalization. Possible treatment options must accomplish drainage of the septic focus in the mediastinum and close the esophageal wall defect or the dehiscent circular staple line of the anastomosis. The traditional conservative method of treating the complication, consisting of systemic antibiotics, conventional thoracic drainage and thoracic irrigation, usually leads to increased duration of hospitalization. Reoperation can achieve adequate debridement and promote conventional drainage but is not a reliable way to achieve fistula closure and is associated with high morbidity and mortality. Several endoscopic treatment options for repair of esophageal anastomotic leakage have emerged to promote fistula closure, including metal clips, fibrin glue, self-expanding metal stents (SEMSs) and self-expanding plastic stents (SEPSs) [6-8]. However, anastomotic leakage remains a challenge, with few available treatment options.

Recently, endoscopic vacuum-assisted closure (E-VAC) has been reported as an effective treatment for closure of perforated diverticula and for intrathoracic and cervical anastomotic leakages in the upper gastrointestinal tract [9-11]. E-VAC involves placing polyurethane sponges through a fistula tube into the infection cavity, followed by application of an external vacuum through a transnasal tube to remove purulent secretions and to induce the formation of granulation tissue. To improve the feasibility of E-VAC drainage, we developed a modified method to accomplish trans-fistula drainage through a simple nasogastric tube without sponge placement or repeated gastroscopy. With simple equipment, we were able to achieve the goals of sufficient drainage, control of the infection source and prevention of further mediastinal contamination with persistent negative pressure.

Herein we report our experience treating anastomotic leakages in five esophageal cancer patients using endoscopic trans-fistula negative pressure drainage (E-TNPD). The objectives of this study were to investigate whether E-TNPD could work as a convenient and reliable method of promoting post-esophagectomy fistula closure by improving trans-fistula drainage and to establish an effective complement to existing therapy.

## Methods

### Patients

Between June and November 2013, five male patients with major post-surgical esophageal leakages who underwent E-TNPD therapy at Beijing Cancer Hospital were enrolled in this study. This study was approved by the Medical Ethics Committee of Beijing Cancer Hospital. Written informed consent was obtained from the patients before the treatment. Three patients suffered from an intrathoracic anastomotic fistula after Ivor–Lewis subtotal esophagectomy, one patient suffered from perforation of a mid-esophageal diverticulum post-esophagectomy and one patient suffered from a cervical anastomotic fistula post McKeown esophagectomy. Tumors were staged according to the TNM classification of the International Union Against Cancer staging system. The characteristics of these inpatients are shown in Table 1.

**Table 1:** Characteristics of five patients who underwent endoscopic trans-fistula drainage

	**Age (years)**	**Pathologically differentiated**	**Surgical mode**	**TNM**	**Neoadjuvant**
Case 1	59	Poorly	Ivor–Lewis^a^	T2N0M0	Yes
Case 2	63	Well	Ivor–Lewis	T3N0M0	No
Case 3	64	Moderately, poorly	Ivor–Lewis	T3N1M0	Yes
Case 4	66	Moderately	McKeown^b^	T1N0M0	No
Case 5	57	Poorly	Ivor–Lewis	T3N2M0	No

^a^Ivor–Lewis esophagectomy: combination of a laparotomy and a right thoracotomy with an intrathoracic subtotal esophagogastrostomy.

^b^McKeown esophagectomy: total esophagectomy through right thoracotomy, laparotomy and neck incision with a cervical anastomosis.

### Diagnosis of leakage

Leakage of the anastomosis was clinically suspected according to manifestations, based on fever or other signs of thoracic infection, characteristics of chest-tube drainage or incision infection. Leakage was clinically diagnosed as contaminated thoracic drainage with precipitant. Subsequently, the leakages were confirmed by esophagography or endoscopic examination. In particular, Case 1 suffered from a diverticulum perforation but not anastomotic fistula. The esophagography on post-operative day (Pod) 9 showed no leakage for him. However, he suffered hemorrhage from the esophageal artery stump and required re-thoracotomy; an intraoperative exploration confirmed a tiny diverticulum perforation, which was neglected during the esophagectomy.

### Traditional conservative treatment

When thoracic infection was confirmed based on contaminated thoracic drainage, a leak was highly suspected and a double lumen tube system was immediately established for irrigation. An intravenous catheter was passed into the lumen of a conventional thoracic drainage tube [12]. Irrigation fluid was introduced via the intravenous catheter lumen, while the larger outer tube had multiple side holes to provide drainage. The thoracic cavity was flushed several times with irrigation fluids containing gentamycin in saline to clear most of the pus. In accordance with the empyema regimen, all patients received intravenous antibiotics and were fed enteral nutrition via a nasojejunal feeding tube.

### Modified negative-pressure-assisted nasogastric drainage

**Figure 1:**
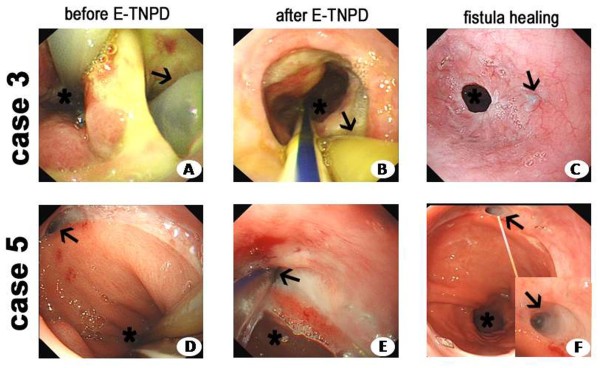
**Endoscopic images of esophageal leakage after esophagectomy and E-TNPD therapy. (A**-**C)** Endoscopic images for Case 3. **(D**-**F)** Endoscopic images for Case 5. **(A)** Endoscopic detection of the esophageal leakage before E-TNPD therapy in Case 3. The defect was obvious and the thoracic tube could be detected endoscopically. The jejunal feeding tube was in the gastric cavity (asterisk). There was a thoracic tube in the para-anastomotic cavity (arrow). **(B)** The nasogastric tube was placed into the para-anastomotic cavity (E-TNPD) and the thoracic tube was subsequently adjusted for appropriate negative pressure. The leakage was obviously recovered. The jejunal feeding tube was in the gastric cavity (asterisk), and the nasogastric tube was in the para-anastomotic cavity (arrow); the cavity had already closed. **(C)** Endoscopic image after complete healing. The anastomosis had some stenosis (asterisk) and the fistula had already healed (arrow). **(D)** Endoscopic detection of esophageal leakage for Case 5. The jejunal feeding tube was in the gastric cavity (asterisk), and the fistula was small (arrow). **(E)** The nasogastric tube was placed into the para-anastomotic cavity (E-TNPD). The jejunal feeding tube was in the gastric cavity (asterisk), and the nasogastric tube was in the para-anastomotic cavity (arrow). **(F)** Endoscopic image of tiny leakage healing. The blind side of the fistula is shown magnified in the inset (arrow).

Based on our previous experience of misplaced nasogastric tubes through fistula sites into the thoracic cavity and on a literature review, we designed the E-TNPD method to clear infected secretions, shrinking the para-anastomotic cavity and eventually allowing closure of the defect. Nasogastric drainage tube placement was performed with a regular 9.5-mm-diameter endoscope (Olympus GIF-H260, Olympus, Tokyo, Japan) (Figure 1). After the esophageal defect was located, its size was estimated and the cavity was flushed with an endowasher via the working channel of the endoscope. Next, a nasogastric silicone tube (DRW-B; BJ Dare Medical Equipment, Baoji, China) was introduced with the grasper forceps into the paraesophageal cavity through the leak site under endoscopic vision. Continuous negative pressure of 25 mmHg was applied using a vacuum pump. The tube itself filled the entire esophageal defect immediately and the negative pressure evacuated fluid secretions, greatly shrinking the fistula tube and decreasing the cavity size. All endoscopic interventions were performed without general anesthesia or sedation.

## Results

### Characteristics of patients

Patient characteristics are summarized in Table 1. We employed E-TNPD treatment for five patients with post-operative esophageal leakage. The patients’ ages ranged from 57 to 66 years. All five patients had undergone surgical excision of esophageal cancer with esophageal-gastric anastomosis. Patients 2 and 3 had intrathoracic anastomotic fistulae. Case 5 had intrathoracic fistulae with concurrent remnant stomach leakage. Case 4 had cervical anastomotic fistulae after a McKeown esophagectomy. Case 1 suffered from mid-esophageal diverticulum perforation after esophagectomy.

### Indications and timing of E-TNPD therapy

For all five patients, we endoscopically verified anastomotic leaks and E-TNPD was successfully accomplished. Two patients underwent endoscopic drainage placement (Case 1 on Pod 52 and Case 4 on Pod 35) after 34 and 28 days of irrigation, respectively, without any progress in fistula healing. Case 1 experienced hemorrhage from an esophageal artery stump and re-thoracotomy confirmed a diverticulum perforation that had been neglected during esophagectomy. On the basis of the successful experiences for the two previous patients, we performed trans-fistula drainage earlier for Cases 3 (on Pod 16) and 5 (on Pod 28). Case 5 developed separate leaks on the circular staple line of the anastomosis and on the body of the gastric conduit. In this patient, we placed two drainage tubes into the different cavities. Case 2 suffered from persistent leakage and concomitant chronic empyema, sepsis, severe continuous diarrhea and subsequent acute renal failure. After hemofiltration therapy, we applied E-TNPD treatment for that patient on Pod 95 (Table 2).

**Table 2:** Endoscopic trans-fistula drainage treatment characteristics

**Case**	**Fistula diagnosed**^**a **^**(Pod)**	**Irrigation duration**^**b **^**(days)**	**E-TNPD performed**^**c **^**(Pod)**	**Duration post E-TNPD**^**d **^**(days)**
1	13	29	52	28
2	11	71	95	26
3	8	8	16	18
4	7	28	35	18
5	7	21	28	81

^a^A fistula was clinically diagnosed if there was contaminated thoracic drainage with precipitant. Subsequently, the leakages were confirmed with esophagography.

^b^Irrigation duration before E-TNPD.

^c^E-TNPD was trans-fistula tube placement and initiation of drainage.

^d^Duration post E-TNPD was the trans-fistula drainage period until tube removal.

### Course of E-TNPD treatment

All patients tolerated negative pressure and did not react with arrhythmia, hemorrhage or hemodynamic complications. No patients complained of increased discomfort resulting from the E-TNPD method or the continuous negative pressure. No serious adverse complications associated with E-TNPD were noted. Once drainage was established and the thoracic infection was controlled, the thoracic drainage became clear within 2 to 3 weeks. We gradually withdrew 5 cm of the trans-fistula tube and placed a nasogastric tube into the gastric conduit lumen as a normal gastrointestinal decompression tube within 1 week of cessation of leakage. The courses of E-TNPD treatment are listed in Table 2. When fistula closure was confirmed by esophagography and pleural empyema was completely controlled, the patient was allowed to begin oral intake.

### Outcome of E-TNPD treatment

**Figure 2:**
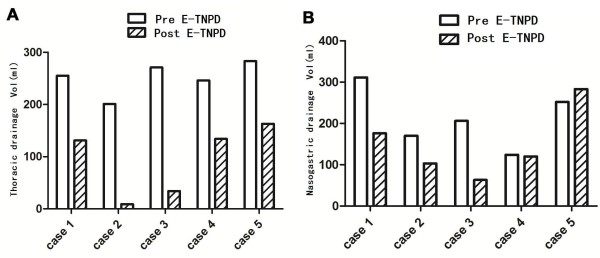
**Drainage volume of thoracic or nasogastric tube before and post E-TNPD. (A)** Before the E-TNPD therapy intervention, the median thoracic drainage volume was 252 ml (range: 201 to 283 ml) with purulent effusion. After institution of the E-TNPD therapy, the median thoracic drainage volume markedly decreased to 94 ml per day (range: 9 to 163 ml). **(B)** E-TNPD promoted resolution of nasogastric tube drainage. Nasogastric tube mean drainage decreased from 202 ml per day (range: 124 to 311 ml) to 115 ml per day (range: 63 to 176 ml) for Cases 1 to 4. For Case 5, the mean nasogastric tube drainage increased from 168 ml to 252 ml, but the mean thoracic drainage decreased from 283 ml to 163 ml. Vol, volume.

Combined with systemic antibiotics and thoracic irrigation, nasogastric trans-fistula drainage promoted fistula closure. In all five patients, complete closure of the fistula was achieved without any procedure-related complications (Figures 1 and 2).

In all five patients, thoracic drainage was found to be contaminated and plural empyema developed, with leakage confirmed on Pods 7 to 13. Patients underwent thoracic irrigation for a median duration of 31 days (range: 8 to 71 days) without signs of improvement. After establishment of E-TNPD, fistula closure was promoted. The median duration of trans-fistula drainage until tube removal for all patients was 34 days (range: 18 to 81 days), with a duration of 18 to 28 days for Cases 1 to 4. Case 5 suffered from multiple separate leaks on the anastomotic circle and the gastric conduit. This patient experienced complete healing in 81 days. With the exception of Case 5, earlier administration of E-TNPD resulted in shorter hospitalization after achieving complete defect closure. All five patients suffered from severe empyema before trans-fistula drainage therapy intervention, with a mean thoracic drainage volume of 252 ml per day (range: 201 to 283 ml), with purulent effusion flushed by thoracic irrigation. After trans-fistula drainage therapy, the mean volume of thoracic drainage decreased markedly to 94 ml per day (range: 9 to 163 ml), and subsequently transformed to normal effusion without pus. Meanwhile, nasogastric tube mean drainage decreased from 202 ml per day (range: 124 to 311 ml) to 115 ml per day (range: 63 to 176 ml) for Cases 1 to 4. For Case 5, mean nasogastric tube drainage increased from 168 ml to 252 ml, but the patient’s mean thoracic drainage decreased from 283 ml to 163 ml.

### Consent

Written informed consent was obtained from the patients before each operation.

## Discussion

Esophageal anastomotic leakage is a challenging condition for both intrathoracic and cervical anastomoses, and there is no current consensus on the best treatment methods. Although several methods, including surgical re-exploration and/or conservative approaches, have been performed, the rate of treatment failure remains high. Because the paraesophageal cavity is difficult to reach with conventional drainage, para-fistula inflammation is difficult to control and leakage defects result in prolonged hospitalization. To address these problems, we developed a modified negative-pressure-assisted trans-fistula method to achieve adequate drainage. This method allows tension-free healing at the leakage site and simultaneously promotes the formation of granulation tissue in the cavity.

A variety of endoscopic techniques have allowed more accurate assessment and management of anastomotic disruption, including clipping, cyanoacrylate glue and SEMS/SEPS. All of these approaches share the primary therapeutic goal of sealing the leakage. Recently, Lenzen *et al*. [10] and Brangewitz and colleagues [11] reported that E-VAC was an effective treatment for leakage and perforations. However, E-VAC can be inconvenient and the method can be improved. Transoral endoscopic placement of a sponge into a leak is technically demanding and the size of the defect must match the sponge. Moreover, patients must undergo repeated gastroscopy for sponge exchange.We developed the E-TNPD method to allow easy access to the infected paraesophageal cavity and rapid removal of infected tissue with persistent negative pressure. In this case series, E-TNPD was a reliable method of draining the para-anastomotic abscess cavity, which is a great challenge with conventional thoracic drainage. It remains uncontroversial that sufficient drainage eradicates local infection, prevents further contamination and controls sepsis. In our cohort, we adopted E-TNPD on Pods 16 to 95 after conservative therapy for 8 to 71 days without signs of improvement. In general, thoracic and nasogastric tube drainage obviously decreased after E-TNPD was established (Figure 1). This decrease indicates that thoracic purulent effusion and reflux infective effusion from the para-fistula cavity were both gradually controlled. For Cases 1 to 4, the total time to achieve defect closure was 18 to 28 days. Even in Case 5, with multiple leaks, defects closed in 81 days. The mean nasogastric tube drainage volume increased for this patient, partly because of the prolonged duration of the persistent nasogastric tube drainage, but the patient’s thoracic drainage markedly decreased. E-TNPD is a reliable and effective method to control contamination in the mediastinum and to promote the recovery process. We also found that generally, the earlier that E-TNPD was instituted, the more effective it was. Cases 1 and 4 had drainage established on Pod 52 and Pod 35, respectively, and their fistulae closed in 28 and 18 days, respectively. Cases 2 and 3 had drainage established on Pod 95 and Pod 16, respectively, and their fistulae closed in 26 and 18 days, respectively. For Case 5, multiple leaks were drained, beginning on Pod 28, and pleural empyema was rapidly controlled, with the fistula closing in 81 days.

In our cohort, we applied E-TNPD for diverse leakages, including anastomotic circle fistula (thoracic and cervical anastomoses), gastric conduit leakage and mid-esophageal diverticulum perforation. In all of these locations, E-TNPD was technically comparatively easy to perform. After E-TNPD, repeated endoscopic examination was not necessary. Stenting requires technical expertise, especially for cervical anastomosis leakage. In addition, the E-TNPD method resulted in a significantly higher closure rate and fewer strictures in patients with intrathoracic leaks compared with the SEMS/SEPS methods [13]. The E-TNPD method can be used to treat leakages over a broad post-operative timeframe. When feasible, E-TNPD was instituted as early as Pod 16 in our cohort. Drainage could also be instituted on Pod 95 for persistent empyema and delayed fistula diagnosis. In addition, Case 1 suffered perforation of a concomitant mid-esophageal diverticulum, Case 4 suffered from a cervical fistula with mediastinal leakage, and Case 5 suffered multiple leakages on the anastomotic circle and the conduit body. All of these patients were treated with E-TNPD and eventually benefited from this treatment. Furthermore, E-TNPD therapy can treat narrow leakages, in which sponge placement would be impossible. E-TNPD should be considered for diverse indications and is acceptable with various timings, fistula types and surrounding situations.

## Conclusions

In conclusion, we found that E-TNPD was a reliable and effective method for managing post-surgical gastroesophageal anastomotic fistula and esophageal perforation. E-TNPD promoted fistula closure and was effective in different leakage locations, post-surgical times, fistula types and surrounding situations. Because there is currently no standard management strategy applicable to all leakages, E-TNPD may be an effective way to complement the existing anastomotic fistula therapy after esophagectomy.

## Abbreviations

E-TNPD: endoscopic trans-fistula negative pressure drainage; E-VAC: endoscopic vacuum-assisted closure; SEMS: self-expanding metal stent; SEP: self-expanding plastic stent.

## Competing interests

The authors declare that they have no competing interests.

## Authors’ contributions

YNL and Y Yan contributed to the preparation of the manuscript. SJL, QW, LJZ and Y Yang participated in the design of the study and performed the statistical analysis. HL and SJL checked the grammar. JFC conceived of the study, participated in its design and coordination, and helped to draft the manuscript. All authors read and approved the final manuscript.
